# Decontamination Efficiencies of Pot-Type Water Purifiers for ^131^I, ^134^Cs and ^137^Cs in Rainwater Contaminated during Fukushima Daiichi Nuclear Disaster

**DOI:** 10.1371/journal.pone.0037184

**Published:** 2012-05-16

**Authors:** Shogo Higaki, Masahiro Hirota

**Affiliations:** 1 Radioisotope Center, The University of Tokyo, Tokyo, Japan; 2 School of Engineering, The University of Tokyo, Tokyo, Japan; Argonne National Laboratory, United States of America

## Abstract

Rainwater was contaminated by a large release of radionuclides into the environment during the Fukushima Daiichi nuclear disaster. It became a matter of concern for Japan when several water purification plants detected ^131^I contamination in the drinking water. In the present study, the decontamination efficiency of two easily obtainable commercial water purifiers were examined for rainwater contaminated with ^131^I, ^134^Cs and ^137^Cs. The water purifiers removed 94.2–97.8% of the^ 131^I and 84.2–91.5% of the ^134^Cs and ^137^Cs after one filtration. Seven filtrations removed 98.2–99.6% of the ^131^I and over 98.0% of the ^134^Cs and ^137^Cs. From a practical perspective, over the fourth filtrations were not needed because of no significant improvements after the third filtration.

## Introduction

On March 11, 2011, an earthquake of magnitude 9.0 occurred with an epicenter near the northeast coast of Honshu, Japan. Subsequently, a massive tsunami hit the eastern coast of Japan. The tsunami reached the Tokyo Electric Power Company’s Fukushima Daiichi nuclear power plant resulting in a large release of radionuclides including ^131^I, ^134^Cs and ^137^Cs into the environment. The radioactive materials were carried by the wind and fell to the ground with dust or rainfall. As a result, radioactive contamination of the environment was not limited to the surrounding area but spread across eastern Japan [1]. In Japan, the source of tap water is largely surface water, such as rivers and dammed lakes. In metropolitan areas such as Tokyo, the sources of tap water are predominantly surface water because of the quantity needed [2]. Therefore, radioactive contamination of rainwater causes radioactive contamination of tap water. After the disaster, radioactive contamination of drinking water was detected at several water purification plants [3]. As an example, on March 22, the concentration of ^131^I was measured to be over 100 Bq/L, which is the Japanese provisional (emergency) criteria for infants, at the Kanamachi Purification Plant, Tokyo [4]. As a result, many people living in the Tokyo metropolitan area hoarded bottled drinking water [5]. As an alternative to bottled water, commercial water purification systems are easy to obtain and can be used to remove harmful substances from contaminated tap water. Commercial water purifiers are widely used in Japan to remove trace amounts of hypochlorous acid from tap water, as mandated by the Japanese Act [6]. Carcinogenic compounds, such as trihalomethanes, are created when hypochlorous acid reacts with impurities. Currently, water purifiers are mainly used to remove these compounds from tap water. Manufacturers have studied the decontamination efficiency of water purifiers with respect to the 13 compounds, such as trihalomethane, identified by the Japanese Act [7]. Commercial water purifiers are classified into three types: pot, attachment and standing. Attachment- and standing-type water purifiers require a pressurization system when purifying rainwater. Additionally, decontamination efficiency of some pot-type water purifiers for the removal off nonradioactive iodine, cesium, strontium, barium and zirconium have also been tested [8]. In contrast, for the radioactive materials released during the Fukushima Daiichi nuclear disaster, the decontamination efficiencies of water purifiers has not previously been tested.

The purpose of this study was to investigate the efficiency with which pot-type water purification systems remove radioiodine and radiocesium from rainwater contaminated by the disaster.

## Methods

**Figure 1:**
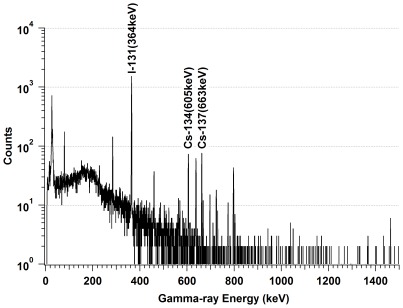
A gamma energy spectrum of rainwater collected in Fukushima City in April 2011. Counting time was 3000 seconds.

One rainwater sample of 2 L was collected in Fukushima City, Fukushima Prefecture (65 km northwest of Fukushima Daiichi) at the beginning of April 2011. The entire rainwater sample was filtered before analysis by a qualitative filter (VWA, Grade No. 413) which retained over 5 µm particle. A 40-mL sample was placed into a polystyrene cylindrical canister (50 mm in diameter, 120 mL in volume) and analyzed using a high-resolution gamma spectrometry system with a coaxial n-type high-purity germanium (HPGe) detector with 25% relative efficiency. A spectrum stabilized 8 K multi-channel analyzer (Princeton Gamma-Tech, MCA8016) and other electronic accessories were coupled with the HPGe detector. The detector was shielded with 10 cm of lead, 0.5 cm of copper and 0.5 cm of acrylic to reduce the background. Figure 1 shows the gamma energy spectrum of the original rainwater sample measured for 3000 seconds. The energy-channel has been calibrated the energy-channel for 0.5 keV per 1 channel with using ^60^Co for 1173 and 1332 keV, ^57^Cr for 122 keV. The peaks of ^131^I, ^134^Cs and^ 137^Cs are clearly observed. The counting efficiency of each gamma peak was estimated from counting efficiency curves prepared using standard solutions certified by the Japan Radioisotope Association (JRIA). The standard solutions of ^131^I, ^134^Cs and^ 137^Cs were provided by JRIA. The concentration of ^131^I was 14.94 kBq/g, ^134^Cs was 0.713 kBq/g and ^137^Cs was 0.741 kBq/g. The certificate identification data of ^131^I was 11-0160, ^134^Cs was 11-0189 and ^137^Cs was 11-0190. ^131^I was determined from the 364 keV energy peaks. ^134^Cs and ^137^Cs were each determined from the 605 and 662 keV energy peaks, respectively. Detection limits were 2.5 Bq/L for ^131^I, 2.0 Bq/L for ^134^Cs and 2.0 Bq/L for ^137^Cs of 40 mL sample in 2000 seconds counting. The detection limits were calculated according to the authoritative guidance [9], [10].

Pot-type water purifier A (Toray Industries, PT302) was equipped with a filter made of activated charcoal and an ion exchanger. The recommended volume limit of the filter was 200 L of tap water. Pot-type water purifier B (Panasonic, TK-CP11) was equipped with a filter made of activated charcoal, ceramics and a hollow fiber membrane. The recommended volume limit of the filter was 240 L of tap water.

Each water purifier was activated by tap water and washed using the rainwater sample. The sample was filtered using qualitative filter paper (VWA, Grade No. 413) which retained over 5 µm particle to remove suspended matter prior to analysis. Subsequently, a rainwater sample of 1 L was filtrated. Upon completion, a small sample of 40 mL was placed into a polystyrene cylindrical canister, and spectrum measurements were taken with the HPGe detector for 2000–3500 seconds. The measured sample was returned to the larger sample and the 1-L sample was filtrated again. This series of operations was repeated seven times.

## Results

The rainwater sample contained ^131^I, ^134^Cs and ^137^Cs. The concentration of ^131^I was 1470±26.5 Bq/L, ^134^Cs was 100±25.3 Bq/L, and ^137^Cs was 129±9.47 Bq/L. The ratio of ^131^I/^137^Cs was 11.5 and ^134^Cs/^137^Cs was 0.858.

**Figure 2:**
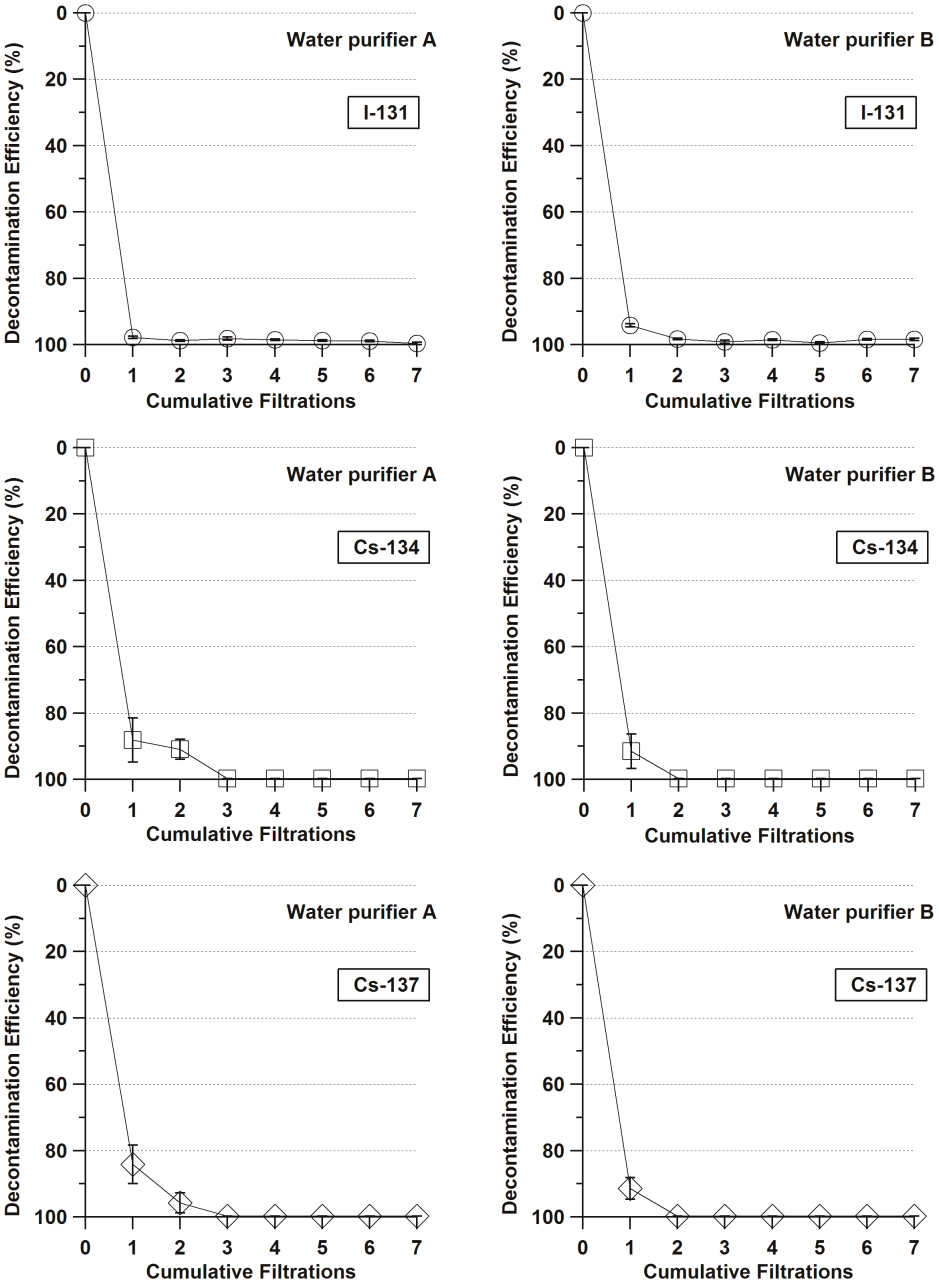
Decontamination efficiencies of pot-type water purifiers, A and B, for each radionuclide.

Figure 2 shows the decontamination efficiency of each water purifier. The decontamination ratios were determined from the following equation:
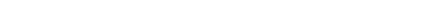
where *T_n_* is the radioactive concentration after n filtrations, and *T_0_* is the radioactive concentration of the unpurified rainwater sample.

For purifier A, the first filtration removed 97.8±0.4% of the ^131^I, 88.1±6.7% of the ^134^Cs and 84.2±5.9% of the ^137^Cs. The decontamination efficiency for ^131^I was improved with additional filtrations, and the maximum efficiency of 99.6±0.3% was achieved after seven filtrations. Additionally, the decontamination efficiencies for ^134^Cs and ^137^Cs were also improved with additional filtrations, and their concentrations were determined to be under the detection limit of the HPGe detector, 2.0 Bq/L, after three purification steps. Therefore, three filtrations removed over 98.0% of the radiocesium.

Purifier B removed 94.2±0.5% of the ^131^I, 91.5±5.2% of the ^134^Cs and 91.4±3.2% of the ^137^Cs after a single filtration. The decontamination efficiency for ^131^I was improved with repeated filtrations; the maximum was 99.5±0.3% after seven filtrations. Additionally, the decontamination efficiencies for ^134^Cs and ^137^Cs were also improved with additional purification steps, and their concentrations were determined to be below the detection limit of the HPGe detector, 2.0 Bq/L, after two filtrations. Therefore, two filtrations removed over 98.0% of the radiocesium.

## Discussion

It has been reported that charcoal is unable to remove iodine reagents (I^−^: iodide ion and IO_3_^−^: iodate ion are known as major chemical species in water) from tap water solutions [11]. In contrast, our results indicate that pot-type water purifiers removed 94.2–97.8% of the ^131^I, and 84.2–91.5% of the radiocesium from contaminated rainwater; the range of values depends on the purifier. These results indicate that commercial water purifiers may be available home solution to the removal of ^131^I, ^134^Cs and ^137^Cs contaminants from water. Additionally, it have been reported that activated charcoal was effective in removing 17% of ^131^I as I^−^ from water [12]. In this way, there are no agreements about whether activated charcoal was efficient to remove ^131^I from water or not. Both I^−^ and IO_3_^−^ are found in rainwater [13]. It seems that I^−^ was easy to be removed by activated charcoal, IO_3_^−^ could not be removed by activated charcoal, ceramics or hollow fiber membranes. By contrast, almost of all of the radiocesium could be removed by two or three filtrations. As Figure 2 shows, there were no significant improvements after the third filtration of both purifiers. From a practical perspective, over fourth filtrations were not needed.

It is known that the chemical forms of gaseous ^131^I commonly found in the exhaust air from a nuclear power plant are CH_3_^131^I, C_2_H_5_^131^I, H^131^I O_4_ and H^131^I O [14]. In liquid effluent samples of nuclear medicine, iodine exists in the dissolved phase primarily as iodide or iodate, but can also form organic species, such as CH_3_I [15]. However, the chemical form of ^131^I from the release of radionuclides during the Fukushima Daiichi nuclear disaster might be different from these forms because the release was not controlled. If distances from the Fukushima Daiichi site or date of rainfall differ from the sample used in this study, the chemical form of ^131^I and amount adsorbed may be different. Therefore, decontamination efficiency for ^131^I using a pot-type water purifier may be different from the ratios obtained in this study.

Cesium released from a nuclear power plant into the environment is transported from the atmosphere to the ground by snow or rain [16]. A cesium ion behaves as a univalent cation (Cs^+^) in water. Cs^+^ is known to bind strongly to a clay mineral having univalent negative charge called the 2∶1 layer silicates in soil, and it is difficult to desorb [17]. One year after the disaster, ^134^Cs and ^137^Cs in soil play a major role in radioactive contamination of the environment. Through a similar reaction, almost all of the Cs^+^ cations in the rainwater were easily adsorbed into the filter and were difficult to desorb.

The present study, with its limited data, may be used to estimate the efficiency of attachment-type or standing-type water purifiers. However, decontamination efficiencies of pot-type purifiers must not be directly applied to other types of purifiers. Their efficiencies must be confirmed with further experiments. Air filters containing charcoal adsorbs gaseous iodine, such as ^131^I_2_ or CH_3_^131^I, from contaminated air such as laboratory to use radioactive iodine [18]. However, it is known that once trapped, iodine desorbs with time [19]. The airflow rate and flow volume may affect desorption. By contrast, it is unclear whether iodine trapped from water by charcoal desorbs with time. If iodine desorbs, decontamination efficiencies of attachment-type and standing-type water purifiers will be less than that of pot-type purifiers.

The present study did not determine the effective water volume and adsorption quantity of pot-type water purifiers. It is expected that for radioactive materials, the decontamination efficiency of a water purifier decreases depending on the filtered water volume and adsorption quantity. The recommended volume limit of a pot-type water purifier is 200–240 L of tap water, and the recommended volume limit for rainwater will be less because rainwater contains more impurities that deteriorate filters more rapidly than tap water. Therefore, the effective water volume and adsorption quantity must be confirmed with further experiments.

A variety of water purifiers are on the market today. If the filters are similarly constructed, comparable decontamination efficiencies can be expected.

## References

[1] Ohta T, Mahara Y, Kubota T, Fukutani S, Fujiwara K (2011). Prediction of groundwater contamination with (137)Cs and (131)I from the Fukushima nuclear accident in the Kanto district.. http://dx.doi.org/10.1016/j.jenvrad.2011.11.017.

[2] Japan Water Works Association (2009). Water Supply in Japan 2009– Japan Water Supply Report 2009.. http://www.jwwa.or.jp/jigyou/kaigai_file/2009WaterSupplyInJapan.pdf.

[3] Ministry of Health, Labour and Welfare website.. http://www.mhlw.go.jp/english/topics/2011eq/watersupply.html.

[4] Tokyo Metropolitan Government website.. http://www.waterworks.metro.tokyo.jp/press/shinsai22/pdf/sokutei/josui_1103_en.pdf.

[5] Consumer Affairs Agency website.. http://www.caa.go.jp/action/kaiken/renho/110325d_kaiken.html.

[6] Ministry of Health, Labor and Welfare (1957). Japanese Water Supply Act..

[7] Ministry of Economy, Trade, Industry (1962). Japanese Household Goods Quality Labeling Act..

[8] Sato I, Kudo H, Tsuda S (2011). Removal efficiency of water purifier and adsorbent for iodine, cesium, strontium, barium and zirconium in drinking water.. J Toxicol Sci.

[9] Cooper J (1970). Factors determining the ultimate detection sensitivity of Ge (Li) gamma-ray spectrometers.. Nucl Instr Methods.

[10] Ministry of Education, Culture, Sports, Science, Technology (2002). Gamma-ray Spectrometry for a Germanium Detector: Radioactivity Measurement Series No.7 Revised..

[11] Watari K, Imai K, Ohmomo Y, Muramatsu Y, Nishimura Y (1988). Simultaneous Adsorption of Cs-137 and I-131 from Water and Milk on “Metal Ferrocyanide-Anion Exchange Resin.". J Nucl Sci Technol.

[12] Goossens R, Delville A, Genot J, Halleux R (1989). Removal of the typical isotopes of the Chernobyl fall-out by conventional water treatment.. Water Research.

[13] Yoshida S, Muramatsu Y, Katou S, Sekimoto H (2007). Determination of the chemical forms of iodine with IC-ICP-MS and its application to environmental samples.. J Radioanal and Nucl Chem.

[14] Tachikawa E, Naotomi M (1971). Chemical Forms of Airborne Radioiodine in the Exhaust Air from Iodine-131 Production Plant.. J Nucl Sci Technol.

[15] Prichard HM, Gesell TF, Davis E (1981). Iodine-131 levels in sludge and treated municipal wastewaters near a large medical complex.. Am J Public Health.

[16] International Atomic Energy Agency (2010). Handbook of Parameter Values for the Prediction of Radionuclide Transfer in Terrestrial and Freshwater: Technical Reports Series 472..

[17] Nakahara O (1998). Fundamentals and Applications of Soil Colloid Science (2) - Structure and Charge Characteristic of Colloid Particles in Soils.. J Jpn Soc Irrigat Drain Reclam Engineer.

[18] Nishizawa K (2009). Technical guideline for safety management of radioactive iodine (part 2).. Jpn J Radiat Saf Manag.

[19] Ito T, Nogawa N, Oohashi K, Nakamura A, Morikawa N (1989). Radioactive Iodine Absorbing Properties of Tetrathiafulvalene.. RADIOISOTOPES.

